# Variability of candidate genes, genetic structure and association with sugar accumulation and climacteric behavior in a broad germplasm collection of melon (*Cucumis melo* L.)

**DOI:** 10.1186/s12863-015-0183-2

**Published:** 2015-03-19

**Authors:** Carmen Leida, Claudio Moser, Cristina Esteras, Ronan Sulpice, John E Lunn, Frank de Langen, Antonio J Monforte, Belen Picó

**Affiliations:** Research and Innovation Center, Department Genomics and Biology of Fruit Crops, Fondazione Edmund Mach (FEM), Via E. Mach 1, 38010 San Michele, Italy; Institute for the Conservation and Breeding of Agricultural Biodiversity (COMAV-UPV), Universitat Politècnica de Valencia, Camino de Vera s/n, 46022 Valencia, Spain; Max-Planck-Institute of Molecular Plant Physiology, Wissenschaftspark Golm, Am Mühlenberg 1, 14476 Potsdam, Germany; Plant Systems Biology Research Laboratory, Department of Botany and Plant Science, Plant and AgriBiosciences Research Centre, National University of Galway, University Road, Galway, Ireland; HMCLAUSE (Business Unit of Limagrain), Station de Mas Saint Pierre, La Galine, 13210 Saint-Rémy-de-Provence, France; Instituto de Biología Molecular y Celular de Plantas (IBMCP), Universitat Politècnica de València (UPV)-Consejo Superior de Investigaciones Científicas (CSIC), Ciudad Politécnica de la Innovación (CPI), Ed. 8E, C/Ingeniero Fausto Elio s/n, 46022 Valencia, Spain

**Keywords:** Melon, Climacteric ripening, Sugar, Germplasm collection

## Abstract

**Background:**

A collection of 175 melon (*Cucumis melo* L.) accessions (including wild relatives, feral types, landraces, breeding lines and commercial cultivars) from 50 countries was selected to study the phenotypic variability for ripening behavior and sugar accumulation. The variability of single nucleotide polymorphisms (SNPs) at 53 selected candidate genes involved in sugar accumulation and fruit ripening processes was studied, as well as their association with phenotypic variation of related traits.

**Results:**

The collection showed a strong genetic structure, defining seven groups plus a number of accessions that could not be associated to any of the groups (admixture), which fitted well with the botanical classification of melon varieties. The variability in candidate genes for ethylene, cell wall and sugar-related traits was high and similar to SNPs located in reference genes. Variability at ripening candidate genes had an important weight on the genetic stratification of melon germplasm, indicating that traditional farmers might have selected for ripening traits during cultivar diversification. A strong relationship was also found between the genetic structure and phenotypic diversity, which could hamper genetic association studies. Accessions belonging to the *ameri* group are the most appropriate for association analysis given the high phenotypic and molecular diversity within the group, and lack of genetic structure.

The most remarkable association was found between sugar content and SNPs in LG III, where a hotspot of sugar content QTLs has previously been defined. By studying the differences in allelic variation of SNPs within horticultural groups with specific phenotypic features, we also detected differential variation in sugar-related candidates located in LGIX and LGX, and in ripening-related candidates located in LGII and X, all in regions with previously mapped QTLs for the corresponding traits.

**Conclusions:**

In the current study we have found an important variability at both the phenotypic and candidate gene levels for ripening behavior and sugar accumulation in melon fruit. By combination of differences in allelic diversity and association analysis, we have identified several candidate genes that may be involved in the melon phenotypic diversity.

**Electronic supplementary material:**

The online version of this article (doi:10.1186/s12863-015-0183-2) contains supplementary material, which is available to authorized users.

## Background

Melon (*Cucumis melo* L.) is one of the most important crops within the Cucurbitaceae family, presenting a high variability in fruit traits among different cultivars, ranging from non-sweet fruits that are harvested before maturity and consumed as vegetables, to sweet fruits with high sugar concentrations that are eaten in salads or as dessert. Melon has been proposed to have an African and/or Asian origin [1], and was subject to intense diversification after domestication, with primary centers of diversity in Central Asia and secondary centers in the Mediterranean basin and Far East countries.

*C. melo* has been divided into two subspecies: ssp. *melo*, and ssp. *agrestis* [2]. Recently Pitrat [3] split these subspecies into 15 botanical groups: ssp. *melo*, which includes *cantalupensis, reticulatus, adana, chandalak, ameri, inodorus chate, flexuosus, dudaim* and *tibish* (later reclassified as ssp. *agrestis* by Esteras et al., [4]), and ssp. *agrestis*, which includes *momordica, conomon, chinensis, makuwa* and *acidulus*. Among these, melon cultivars belonging to the *cantalupensis, reticulatus* and *inodorus* groups are economically the most important (e.g. cantaloups, western shippers, galias, ‘Piel de Sapo’ and honeydew).

Melon fruits display a broad range of phenotypic variation. Melon fruit weight varies from a few grams to several kilograms (fruits up to 35 kg have been reported), and the shape may be round, oblate, ovate, elliptical or extremely elongated [5-8]. A huge variability also exists for other characteristics associated with fruit quality, such as flesh color, sugar content and aroma [9]. Different combinations can be found, varying from the non-sweet non-aromatic fruits of cultivars from the *flexuosus* group to the sweet and aromatic *cantalupensis* melons [10].

Differences in sugar content among cultivars mainly reflect differences in sucrose accumulation [8]. Sucrose accumulation is controlled by a major gene [9] that explains the main differences between sweet and non-sweet cultivars, although multiple minor quantitative trait loci (QTLs) for sugar accumulation have also been reported [11]. The metabolic pathway of sugar metabolism in melon fruit has been investigated in several studies [9,12]. Melon, like other cucurbits is a symplastic phloem loader that synthesize raffinose and stachyose from sucrose in specialized intermediary cells in source leaves. These two raffinose-family oligosaccharides (RFOs), plus sucrose, are translocated from source leaves to the developing melon fruit. After phloem unloading in sink organs, the RFOs are hydrolyzed by acid and neutral α-galactosidases (AAG, NAG), producing sucrose and galactose. The latter is phosphorylated by galactokinase and then converted to glucose 6-phosphate, which can either be respired or used to synthesize sucrose via sucrose-phosphate synthase (SPS) and sucrose-phosphate phosphatase (SPP). Sucrose unloaded from the phloem can be hydrolyzed in the apoplast by cell wall invertase (CIN). The resulting hexose sugars (glucose and fructose) are imported into cells by monosaccharide transporters, phosphorylated by hexokinase (HXK) and fructokinase (FK) and used for respiration or sucrose resynthesis. Sucrose can also be unloaded symplastically. Within the cell, sucrose can be catabolized in the cytosol by sucrose synthase (SUS) or neutral invertase (NIN), or imported into the vacuole for storage or hydrolysis by vacuolar acid invertase (AIN), with potential regulation of the latter by invertase inhibitor proteins (INH). During early fruit development, sucrose catabolism predominates, as the carbon and energy derived from sucrose are needed for growth-related processes. As the fruit develops, more and more sucrose is stored rather than respired, and this transition from sucrose catabolism to storage is characterized by loss of AIN activity. Vacuolar processing enzymes (VPE) participate in protein maturation in the vacuole and are also implicated as factors in sugar storage. For example, a reduction in the rate of the proteolysis of vacuolar invertases can lead to their accumulation and modify sugar metabolism and accumulation [13]. Some of the genes encoding sugar metabolizing enzymes and VPEs have been mapped in melon using specific biparental populations [11,14-16].

Melon also comprises broad genetic variation for ripening behavior, with climacteric and non-climacteric varieties. Typical climacteric melons are found within the *cantalupensis* group. These exhibit a distinct peak in respiration and ethylene production at maturity, and generally have a short ripening time and rapidly deteriorate in quality after harvest [17]. In contrast, melons from the *inodorus* group are unable to produce autocatalytic ethylene [18] and, in general, ripen more slowly and have a longer postharvest shelf life [19]. This diversity in ripening behavior makes melon an ideal subject for investigating the physiological and genetic basis for differences between ethylene-dependent and ethylene-independent fruit ripening. There have been several studies of the inheritance of climacteric ripening behavior in melon. Perin et al. [17], investigated the segregation of the formation of the abscission layer and ethylene production in a climacteric x non-climacteric cross. Both traits were controlled by two independent *loci* (*Al-3* and *Al-4*) in linkage groups (LG) VIII and IX, and four further QTLs in LGs I, II, III and XI. In a collection of near isogenic lines (NILs) derived from two non-climacteric genotypes [20-22], one NIL showed climacteric behavior [14]. This NIL carried two introgressions in LG III and LG VI, and both of them had QTLs involved in climacteric ripening (*ETHQB3.5* and *ETHQV6.3*, respectively), which interacted epistatically [23]. Fine mapping studies narrowed down the position of *ETHQV6.3* to a 4.5-Mbp physical region of the melon genome [23].

Ethylene affects the expression of many ripening related genes, in both climacteric and non-climacteric fruits, but expression of other genes is ethylene independent even in climacteric fruits [24-26]. Most of the research based on the regulation of ripening has been focused on the climacteric tomato fruit. The deciphering of the ethylene biosynthetic pathway, including the isolation of the two key enzymes, 1-aminocyclopropane-1-carboxylate (ACC) synthase and ACC oxidase (ACS and ACO) [25], represented substantial advances in our understanding of the role of this hormone in tomato ripening. Further insights came from identification of components of the ethylene perception and signal transduction pathways. These includes ERS (ETHYLENE RESPONSE SENSOR) and ETR (ETHYLENE RESPONSE), which encode membrane proteins involved in signal reception, RTE1 (REVERSION TO ETHYLENE SENSITIVITY 1) that might be involved in negative feedback of ethylene responses, and CTR1 (CONSTITUTIVE TRIPLE RESPONSE 1*)*, which encode a Raf-like kinase that negatively regulates the downstream ethylene response pathway. Also transcription factors such as ERF (ETHYLENE RESPONSIVE FACTOR), EIN (ETHYLENE INSENSITIVE), EIL (ETHYLENE-INSENSITIVE LIKE), and EBF (EIN3-BINDING F-BOX) are involved in ethylene responses [26-28].

In tomato, mapping of mutants showing defects in fruit ripening, such as *ripening inhibitor (rin;* also called *MADS-RIN), non-ripening (nor,* also called *NAC-NOR)*, *colorless non-ripening (Cnr,* also called *SPL-CNR)* and *NR* (*never ripe*) revealed that all the underlying lesions were in transcription factor genes [29-31]. Other transcription factors shown to be involved in ripening include SlHB-1, ETO1, E8 and E4/E8BP. SlHB-1 is a HD-zip homeodomain protein that interacts with ACO1, decreases ethylene synthesis and delays ripening [32]. ETO1 (ETHYLENE OVERPRODUCER 1) is a negative regulator of ethylene ACS type2 [33]. E8 is induced in mature fruits in response to ethylene, although its precise function is still not well defined [34]. E4/E8 binding protein is a protein that interacts with E8 promoter sequences, acting as a positive regulator during fruit ripening [35]. Despite the discovery of the key factors in fruit ripening [36] and interactions between them [37], much remains to be learned before we have a complete understanding of this complex process.

An array of genomic and genetic tools has become available in the last few years for melon research, including genetic maps [11], microarrays [38], TILLING and EcoTILLING platforms [39,40], new mapping populations such as NILs [41] and double haploid lines (DHLs) [42], deep transcriptomic sequencing data [43,44], and a complete genome sequence [45]. These tools are now being deployed to investigate the physiological and genetic basis for agronomically important traits in melon, including fruit ripening and sugar content.

The huge genetic diversity of the species has been studied with different molecular markers [46]. However, despite the availability of massive collections of SNPs, these are still underexploited. Blanca et al. [44] created the most complete version of the melon transcriptome to date, using a combination of expressed sequence tags (ESTs) from Sanger sequencing and next generation sequencing methods, e.g. 454 (Roche) and SOLID (Life Technologies Inc). The resulting database contains thousands of *in silico* identified SNPs, representing the largest collection existing for melon (www.melogene.net).

In an attempt to study the variation of genes involved in sugar metabolism and the ripening process, we searched the Melogene database for SNPs located in a set of candidate genes involved in these processes. We used this set of SNPs, along with reference SNPs evenly distributed in the genome, to genotype a set of 175 melon accessions, including commercial varieties, landraces, and wild or feral melons from over 50 countries, representing the wide diversity within the species. We report the variability of a set of genes involved in ripening behavior and sugar accumulation in melon fruit, providing a framework for studying the putative role of those genes in the diversification of the species, and to associate allelic variants with the phenotypic differences within the germplasm.

## Results

### Germplasm population structure

**Figure 1:**
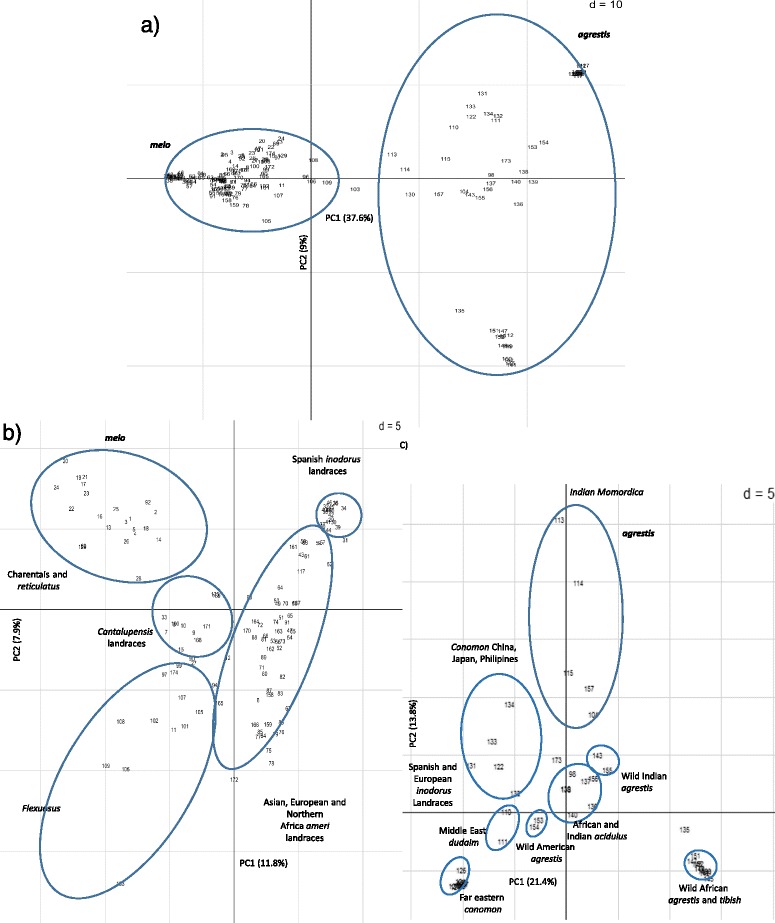
**Principal Component Analysis (PCA) based on 210 SNP markers distributed through the melon genome and in candidate genes for quality traits. a)** the PCA analysis for all the germplasm collection. **b** and **c)** *C. melo* ssp. *melo* and *agrestis* considered separately.

The genetic diversity of the whole germplasm collection (Additional file 1) based on SNP variability (Additional file 2) was analyzed using principal component analysis (PCA) and STRUCTURE. The PCA approach showed a clear differentiation between the two subspecies, *melo* and *agrestis* (Figure 1a), so in order to investigate more subtle genetic structure, we performed the PCA for each subspecies separately (Figure 1b and c). Within ssp *melo* (Figure 1b)*,* the PC1 axis separated *inodorus* from *cantalupensis* and *reticulatus* cultivars. Taking together the first two PC dimensions (11.8% and 7.9% of the total variance for PC1 and PC2 respectively), a group of Spanish *inodorus* landraces (located in the upper-right part of the plot) is clearly differentiated from a group that includes other Spanish landraces and *inodorus* and *ameri* cultivars from Eastern Europe, Asia and North Africa (located in the center/lower-right part of the plot). The *cantalupensis* types could also be split into two groups: modern ‘Charentais’ and *reticulatus* cultivars in the upper-left area, and older cantaloup landraces in the central part of the plot. The non-sweet *flexuosus* types were grouped together, but clearly separate from all the sweet melons.

For ssp. *agrestis* (Figure 1c), two groups could be distinguished along the PC1 axis (21.4% of the total variance): African wild *agrestis* accessions with *tibish* varieties, and *conomon* types that originate from the Far East. The other accessions of this subspecies, including Indian *momordica*, wild Indian *agrestis*, Indian and African *acidulus*, Middle East *dudaim* and additional Far East *conomon* were distributed between the two extreme populations. Some wild American melons, likely representing African or Asian introductions, were also found between those populations. Along the PC2 axis (13.8% of the total variance), the Indian *momordica* accessions could be distinguished from the rest. This distribution of the genetic variability in the PCA space supports previous observations, indicating that Far East *conomon* varieties represent one extreme within the overall genetic distribution of melons [5,47], and that the African and Indian wild melons are genetically distinct [4].

**Figure 2:**
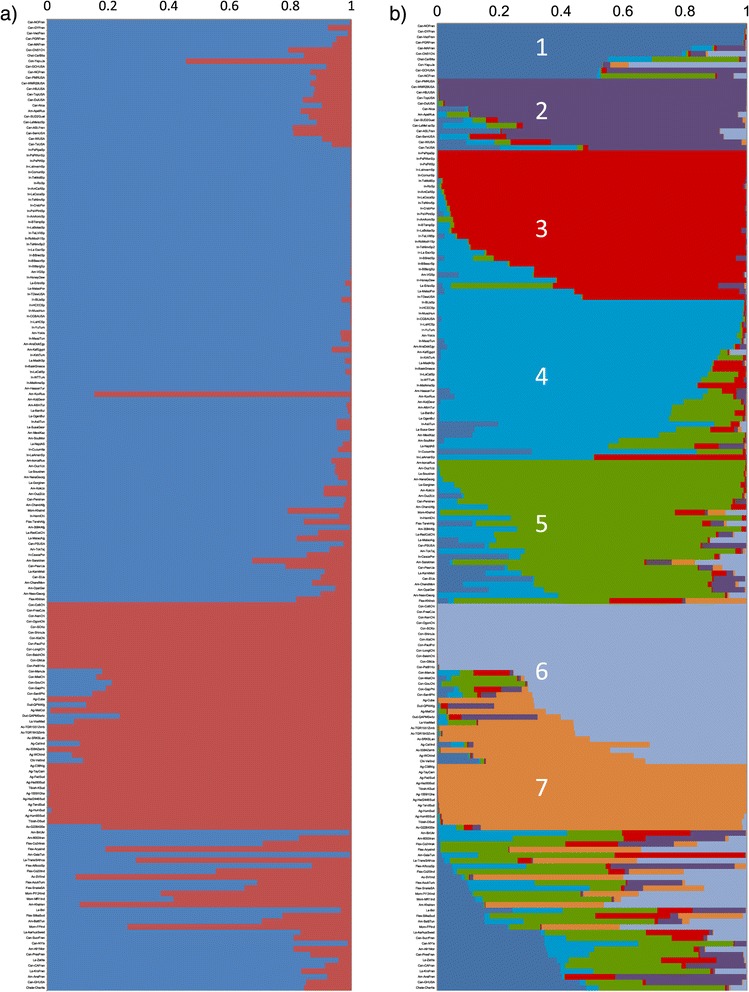
**Inferred population structure of the collection using STRUCTURE** **[** 75 **].** Each accession is represented by a line that is partitioned into coloured segments in proportion to the estimated membership in the corresponding populations. **a)** Best K choice based on the ΔK method K = 2; blue line represents *melo* subspecies and red line *agrestis.* **b)** second best choice K = 7; Dark blue line represents ‘Charentains’ group (1), purple line *reticulatus* (2), red line *Spanish Inodorus* accessions (3), light blue line a mixture of *inodorus* and *ameri* (4), green line mostly *ameri* (5), light purple line *conomon* (6) and orange line *African agrestis* (7). Abbreviations: *Can = cantalupensis, In = inodorus, Am = ameri, Flex = flexuosus, Cha = chate, Dud = dudaim, Con = conomon, Mom = momordica, Chi = chito, Tibish = tibish, Ag = agrestis. La = landraces*. Last three letters indicate the country of origin.

Analysis with STRUCTURE following the Evanno ∆K approach [48] to determine the number of populations gave a maximum value when K = 2, with lesser maxima for K = 5 and K = 7 (Additional file 3). The most strongly supported division into two subpopulations (K = 2) reflects the classification into two subspecies, *agrestis* and *melo* (Figure 2a), which is supported by most previous molecular studies [4] and the initial PCA of our complete dataset. Further resolution into seven sub-groups (K = 7) was consistent with groupings based on geographical origin and fruit characteristics (Figure 2b). Five of these groups belong to ssp. *melo* and two to ssp. *agrestis*, with a small number of accessions in a mixed group that was not clearly resolved. Within subspecies *melo,* the *cantalupensis* varieties split into two groups. The first includes mostly French ‘Charentais’ varieties (population 1, dark blue line in Figure 2b), such as ‘Vedrantais’ and ‘Nantais Oblong’. The second includes *reticulatus* melons (population 2, dark purple line in Figure 2b), with both commercial cultivars and breeding lines, most of which have an American origin (e.g. ‘Top Mark’, ‘Dulce’, ‘PMR 45’). Some other commercial American *reticulatus* cultivars (e.g. ‘Golden Honey’, ‘Golden Champlain’) seem to be a mixture of Charentais and *reticulatus* populations. Some other cultivars from different origins were also included within one of these groups. Thus, the Japanese ‘Yamato Purinsu’ and the Chinese ‘China 151’, both considered *makuwa* types, were included with the French group although they showed some commonality with the *conomon* population (population 6, light purple line in Figure 2b). These two cultivars have been used in breeding commercial melons due to their high fruit quality and resistance to viruses [49].

Population 3 (red line in Figure 2b) contains mostly *inodorus* varieties, especially Spanish and Portuguese casaba melons, commercial cultivars and landraces of the market classes ‘Piel de sapo’ (e.g. ‘Pipa de oro’, ‘Piñoncillo’, ‘Piñonet’ , ‘Verde Pinto’), ‘Amarillo’ (e.g. ‘Amarillo Oro’ , ‘Caña Dulce’), ‘Tendral’ (e.g. ‘Mollerusa’ , ‘Negro de Invierno’), ‘Rochet’ (e.g. ‘Mochuelo’), and ‘Blanco’ (e.g. ‘Crabranco’, ‘Tempranillo’). Other singular Spanish landraces that show some traits of climacteric ripening including aroma and flesh softening (e.g.‘Hilo carrete’ , ‘Madura Amarilla’ , ‘Amarillo Manchado’ , ‘Calamonte’ , and some ‘Blanco’ types), are not classified in the common market classes. These formed a separate population (Population 4 light blue in Figure 2b) along with *inodorus* varieties from Northern Africa, Eastern Europe and Western Asia (e.g. ‘Muscatello’ , ‘Maazoon’ , ‘Cassaba golden’ , ‘Kirkagac’ , ‘Yuva’). This population also included some Turkish, Russian, Israeli and Egyptian varieties that belong to the highly variable *ameri* group (e.g. ‘Hassanbey’, ‘Kuvinska’ , ‘Ananas Yokneam’ , ‘Ananas Dokki’).

A group of accessions from Iran, Uzbekistan and Russia (e.g. ‘Korca’ , ‘Souski’ , ‘Ouzbeque’ , ‘Gorgab’ , ‘Persia’), which mostly belong to the *ameri* pool, formed the last population of subspecies *melo* (population 5 green line in Figure 2b). This population showed the highest genetic variability (gene diversity = 0.22), whereas population 3 showed the lowest (gene diversity = 0.09) (Additional file 4). Most of the landraces from Eastern and Central Asia or the Middle East, show high levels of admixture with one or more ssp. *melo* populations. There seems to be a continuous degree of overlap between population 4 and 5 found in most *ameri* landraces, such as ‘Koljonitza’ and ‘Mucha Nesvi’ from Georgia, ‘Altinbas’ from Turkey, ‘Chandalak’ from Rusia and Mongolia, ‘Tokash’ from Tajikistan or ‘Mestnaia’ from Kazajistan.

Within the *agrestis* subspecies, two clear populations could be distinguished, in agreement with the PCA. Accessions belonging to different types of the *conomon* group (*makuwa*, *chinensis* and *conomon*) from Far Eastern countries (China, Japan, Korea, and Philippines) were grouped in population 6 (light purple in Figure 2b). Wild African *agrestis* from Ghana, Nigeria and Sudan, as well as the cultivated *tibish* from Sudan, which is considered to represent a first step of domestication in Africa, are clearly separated from *conomon* melons (population 7 orange color in Figure 2b). Most *agrestis* accessions from India and America showed a clear mix between these two populations, supporting a common ancestral origin.

None of the remaining accessions belonged to any specific population, but exhibited a high degree of admixture between populations of both subspecies. For example, the *flexuosus* varieties from Mediterranean and Middle East countries, traditionally included in the subspecies *melo,* show mixed *agrestis*-*melo* patterns. A similar situation is observed in the *dudaim* (mix of *conomon* and *reticulatus*) and *momordica* varieties (mix of the two *agrestis* populations with *ameri* and *cantalupensis*). *Momordica* has been traditionally assigned to the *agrestis* subspecies, but our results reflect the proximity of these *agrestis* varieties to the *melo* group. The subgroups demarcated by the PCA coincide with the sub-populations identified by STRUCTURE analysis, corroborating division of the overall population into the designated groups.

### Variability in fruit traits and ripening behavior

All accessions have been characterized for different fruits traits: fruit weight, flesh color, sugar and malate content, and also for traits related to climacteric behavior, such as abscission layer formation, fruit detachment, and flesh firmness (Additional file 5). In general, the traits showed a continuous distribution, fitting or approximating a normal distribution. Variability within and between sub-populations was observed for most traits. All *cantalupensis* and *reticulatus* cultivars of the structure populations 1 and 2 had fruits with medium size (average fruit weight ± sd = 816 ± 348 g), mostly with orange flesh, with medium to high sugar content (°Brix = 9.5 ± 1.5 and 9.1 ± 1.2 in VCO and COMAV trials respectively, and average sucrose content = 97 ± 33 μg/g fresh weight). These accessions also clearly show strong climacteric behavior, most with a fully formed abscission layer and fruit detachment, whereas the Spanish group of *inodorus* landraces (population 3) had bigger fruits (fruit weight = 1,027 ± 315 g), with green, white or cream flesh, with higher sugar content (°Brix = 11.1 ± 1.1 and 10 ± 1.7 and average sucrose = 134 ± 44 μg/g fresh weight), and were mostly non-climacteric with no abscission layer or fruit detachment.

A higher variability in most traits was observed among accessions assigned to sub-populations 4 and 5. These developed medium to large sized fruits (fruit weight = 1,017 ± 425 and 975 ± 554 g, for populations 4 and 5 respectively), with green, white, yellow, cream or light orange flesh, and variable sugar content (°Brix = 9.1 ± 2.0 -7.1 ± 2.3 in the VCO trial and 8.0 ± 1.8-7.0 ± 1.9 at COMAV, and sucrose content = 114 ± 42 and 60 ± 46 μg/g fresh weight, respectively in both populations). The ripening behavior was also variable, including some Spanish landraces with certain climacteric behavior, typical non-climacteric *inodorus* and some *ameri* cultivars with different degrees of climacteric behavior (from no to full fruit slip).

Other ssp. *melo* accessions (*cantalupensis*, *reticulatus*, *ameri*, and *inodorus*, and other landraces) that show admixture of two or more of these sub-populations (populations 1 to 5) were also variable for fruit size and flesh color. Most had medium to high sugar content, but exhibited different degrees of climacteric behavior, ranging from clearly non-climacteric cultivars to some fully climacteric cantaloups and *ameri* accessions, with a wide range of intermediate behaviors (Additional file 5).

Within the subspecies *agrestis*, sub-population 6, which includes *conomon, makuwa* and *chinensis* types, showed small fruits (fruit weight = 464 ± 266 g), mostly with green or white flesh, and wide variation in sugar levels (°Brix = 7.5 ± 3.1-7.2 ± 2.1 in VCO and COMAV, and sucrose content = 78 ± 43 μg/g fresh weight). They also showed different ripening behaviors, ranging from non-climacteric to weakly climacteric. Similar variation in ripening behavior was also observed in some wild African *agrestis* melons (population 7) that turn yellow during ripening and show signs of forming an abscission layer. However, this population was quite uniform for fruit size, flesh color and sugar content, developing very small, green-fleshed, non-sweet fruits (fruit weight = 31 ± 30 g, °Brix = 6.3 ± 3.8-6.9 ± 2.6 in VCO and COMAV, and sucrose content = 37 ± 53 μg/g fresh weight). Accessions included in the admixture *melo-agrestis* group, consisting of *momordica*, *dudaim*, *flexuosus* and *chate* cultivar types, generally had little or no sugar, and showed weak to strong climacteric behavior (Additional file 5).

### Variability in candidate genes

Out of a total of 251 SNPs assayed, 210 were polymorphic in the population. Of these, 37 were located in ethylene metabolism or cell wall related genes and 27 in sugar metabolism candidates (Additional file 2). Variability at ethylene and cell wall related SNPs (gene diversity = 0.37) is slightly higher than at sugar related SNPs (gene diversity = 0.31), and similar to reference SNPs (gene diversity = 0.41) (Additional file 6). SNP variability among the ethylene and cell wall related SNPs had a higher weighting than sugar related SNPs in the first component of the PCA (PC1 in Figure 1a), which separates the two subspecies (Additional file 7). This indicated that variability in the ethylene and cell wall related genes made a greater contribution to the sub specific genetic structure of the collection than the sugar-related genes. However, SNPs among all three groups of genes appeared to make similar contributions to the separation in PC2, which mainly distinguishes African *agrestis* from the others. Interestingly, the candidate genes for ethylene metabolism and cell wall have a larger contribution in the population differentiation found with STRUCTURE than candidate genes for sugar content (Additional file 8).

### Relationship between candidate gene variation and cultivar classification

Some SNPs in sugar-related genes have an allele specific for one of the groups defined by STRUCTURE (Additional file 9). For example, the C/T SNP in *CmINH1*, which causes a non-tolerated (according to SIFT) A126V amino acid substitution in invertase inhibitor 1 (CmINH1.1), appears only in the French ‘Vedrantais’ cultivar and some closely related ‘Charentais’ melons, and in the *makuwa* ‘Yamato Purinsu’ and ‘China51’ genotypes that are known to have been used in cantaloup breeding [49]. All these cultivars belong to the STRUCTURE-defined sub-population 1 (Figure 2). They are sweet climacteric varieties that show a decline in sucrose content upon harvest. Invertase inhibitors potentially play a role in reducing invertase activity, thereby allowing sucrose to accumulate in the developing fruits. *CmINH1* is the mostly highly expressed of the three invertase inhibitors at the onset of sucrose accumulation in the *reticulatus* ‘Dulce’ genotype [12]. Another SNP in the 3´-UTR region of the same gene, CmINH1.4, is almost exclusively present in wild *agrestis* types and *tibish* from Sudan. Similar SNPs in other invertase inhibitors, such as a C/T mutation that causes a tolerated P31S substitution in CmINHLIKE2.1, have a slightly less restricted distribution, occurring in other non-sweet, wild African *agrestis* types as well as *agrestis* and *tibish* from Sudan (all in STRUCTURE sub-population 7) (Additional file 9). The same allelic distribution is found in a 3´-UTR mutation in the fructokinase gene (*CmFK3*), in a non-tolerated C/T (F9L) mutation in the vacuolar processing enzyme CmVPELIKE2.3, and in a synonymous C/T (Q721Q) change in *CmSUS3* encoding one isoform of sucrose synthase (CmSUS3.1). Similarly, the non-tolerated C/A (L267I) mutation in *CmSPP1* (CmSPP1.1) is present only in a few African wild types, *tibish* cultivars and in some African *acidulus. CmSUS3* is the mostly highly expressed *SUS* gene during the sucrose accumulating period in *reticulatus* melons, and *CmSPP1* increases its expression during ripening [12]. Most of these genes make a major contribution to PC2 in Figure 1a (Additional file 7), which mainly separates African *agrestis* from other melons. Some of these genes also make a substantial contribution to the genetic differentiation among groups (high Fst and R2 from AMOVA) (Additional file 8).

Other SNPs in sugar-related candidates had more balanced frequencies for both alleles, and allelic variation was found within specific populations inferred by STRUCTURE and within the admixture group. Some of these SNPs showed an interesting pattern among sucrose accumulating and non-accumulating accessions. For example, most non-sweet or low-sugar genotypes in several populations (African, Indian and American *agrestis*, *tibish*, *acidulus*, *flexuosus*-*chate*, *dudaim*, *conomon* and *momordica*) had a non-tolerated T/C (S173P) mutation in *CmAIN2* (CmAIN2.3), which also appears in some exotic medium sugar *ameri* and cantaloups (e.g. ‘Chandalack’, ‘Pearl’, ‘Earl favourite’, ‘Seminole’, ‘Persian’), but is absent in the other sweet genotypes. Most cantaloups and all *inodorus* had the alternative allele (Additional file 9). *CmAIN2* encodes an acid invertase that is expressed in young fruits but not at maturity [12,50]. If invertase activity reflects the change in expression at the transcript level, the enzyme is likely to contribute to sucrose hydrolysis in young fruit but then decrease in activity as the fruit develops, allowing sucrose to accumulate in mature fruit.

There are SNPs in the 3´-UTRs of two invertase inhibitor genes (CmINH1.3 and CmINH3.1), and in each case, one allele tends to occur more frequently in sweet genotypes (*inodorus, cantalupensis* and *ameri*), although also present in a few low sugar *momordica*, *dudaim* and *chate* types. Also the CmINHLIKE2.4 SNP (C/T giving rise to a tolerated S137A substitution), shows a variation pattern similar to that of *CmAIN2,* with most of the non-sweet *agrestis* and *melo* genotypes (*agrestis, tibish, acidulus, momordica, conomon, dudaim, flexuosus, chate*) sharing the same allele as a few cantaloups. One synonymous mutation in the coding region of *CmVPELIKE3* (CmVPELIKE3.2) and one 3´-UTR change in *CmAAG2* (CmAAG2.1) could also be related with sugar content. The expression studies by Dai et al [12] suggested that the acid α-galactosidase encoded by the *AAG2* gene plays a role in hexose production only in the early stages of fruit development in the ‘Dulce’ *reticulatus* genotype. This genotype has the C allele, more common in the sweet genotypes. Most of these mutations are located in the sugar candidate genes with the highest contributions to the PC1 in Figure 1a (Additional file 7), which separate both subspecies. QTLs for sugar accumulation have been reported previously in the genomic regions linked to some of the discussed genes, especially *CmAIN2* and *CmVPE-LIKE3* (LG IX), and *CmAAG2* (LG X) [11].

The ripening-related gene candidates that contribute most strongly to differentiation between the subspecies (such as the *CNR, AtEIN3*, *CmACO3* and*CmERF3* genes linked to ethylene metabolism, and the cell wall related gene *CmEXP3*) (Additional file 7) have different alleles in ssp. *melo* (sub-populations 1-5) *versus* ssp. *agrestis*, (sub-populations 6 and 7), and are almost fixed within the respective subspecies (Additional file 9). This echoes the pattern seen for sugar related genes, where some of the STRUCTURE sub-populations carry a specific allele for some of the SNPs. For example, the rare alleles of two SNPs, CmEIN3LIKEex2 (a G/T change that causes a tolerated L36V substitution) and AtEIN3ex2 (a C/T synonymous substitution of I499I), both located in the same melon gene MELO3C015633 (an EIL3 transcription factor involved in ethylene signaling [51]), were fixed in the population of *inodorus* (sub-population 3), mostly composed of clearly non-climacteric genotypes. The *cantalupensis* and *reticulatus* populations, which are both highly climacteric, have the alternative allele, which is also more frequent in all of the remaining populations composed of genotypes with different degrees of climacteric behavior.

Other SNPs are also differentially distributed between highly climacteric *cantalupensis* and *reticulatus* (sub-populations 1 and 2) and non-climateric *inodorus* (sub-population 3). However, in this case the allele associated with climacteric behavior is also fixed in the *conomon* population (sub-population 6), while the allele that is more commonly associated with non-climacteric behavior appears in populations 4, 5 and 7. The latter three populations show variable levels of climacteric behavior. These SNPs include a tolerated C/G mutation (H121D) in the coding region of the cell wall related gene *CmXTH5*, a SNP in the 3´-UTR of *CmACO3*, and a synonymous T/G mutation (G171G) in *CmERF2*. All these SNPs contributed to the genetic differentiation of STRUCTURE populations (Additional file 7).

### Linkage disequilibrium

**Figure 3:**
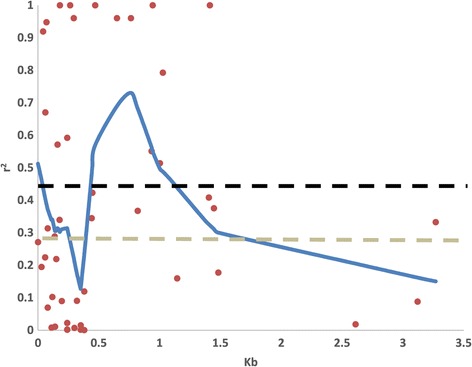
**Linkage disequilibrium (r** ^**2**^ **) versus physical distance (kb) in the accessions considered for the association linkage analysis.** LD extension is shown for a subpopulation of melons, excluding African *agrestis*, and was used in the association analysis for fruit quality traits. The false discovery rates p < 0.05 and p < 0.01 are indicated with black and grey dashed lines respectively. Curves were fitted by second degree LOESS.

Linkage disequilibrium (LD) was studied in order to assess the possible degree of linkage between SNPs associated with the studied traits (see below) and real causal SNPs. Wild melon accessions were excluded because the fruits from these accessions differed in too many respects from fruits of cultivated accessions, making direct comparisons difficult if not impossible. Intra-chromosomal LD showed a rapid decay within a physical distance of less than 0.5 kbp, but increases from 0.5 to 1 kbp and then decreases rapidly with larger distances (Figure 3). This is similar to the results of Esteras et al. [4], who found that the LD extension decayed at less than 3 kbp. Thus, in the current data set, causal SNPs are expected to be very closely linked to the SNPs that are significantly associated with trait variation.

### Association analysis for sugar and organic acid content

Association analysis was investigated using either a general linear model (GLM) or mixed linear model (MLM) approach, with the latter method being used to correct for the effect of genetic structure. GLM analysis gave a high number of significant associations (Additional file 10), including a consistent association of SNPs in sugar candidates (such as CmINHLIKE2.1 and CmAIN2.3) with Brix and sucrose content, whereas MLM analysis provided only a few significant associations (Table 1). The larger number of associations obtained by GLM is attributable to the strong genetic structure in the current germplasm sample, so we focussed on the MLM results, which are likely to be more robust.

**Table 1:** **Markers associated with melon fruit traits of interest**

	**Marker**	**Transcription model**	**Chr**	**locus_position**	**R** ^**2**^	**p value**	**Nucleotide position**	**SNP**	**AA change**
***Climacteric behavior***									
ABCISSION LAYER	MLO65044.1	MELO3C005044	12	3625217	0.111	5.475E-05	1481	C/T	L454L
***Firmness***									
FLESHFIRMESSVCO	PSI_41-B07	MELO3C021426	11	22820373	0.134	1.996E-05	593	C/T	F167F
***Sugar***									
BRIXVCO	SlERF1	MELO3C011287	3	22138473	0.133	2.598E-05	208	C/T	N62N
BRIXCOMAV	CMPSNP711	MELO3C021106	1	26308138	0.103	0.0001498	2382	C/T	3´UTR
BRIXCOMAV	SlERF1	MELO3C011287	3	22138473	0.131	1.839E-05	208	C/T	N62N
Sucrose	SlERF1	MELO3C011287	3	22138473	0.112	7.56E-05	208	C/T	N62N
Sucrose	SlERF3	MELO3C002624	12	20075012	0.132	3.144E-05	481	A/G	Q138Q
***Organic acid***									
malic acid	CmINHLIKE2.2	MELO3C017187	2	22908768	0.101	0.0001873	245	C/T	S60S
malic acid	AtEIN3ex4.2	MELO3C019931	3	17934192	0.084	0.0003247	1464	C/T	F375F
malic acid	CMPSNP677	MELO3C009586	4	26788563	0.088	0.0002695	2976	C/G	3´UTR
***Fruit color***									
C_FLESH	CmXTH5	MELO3C012004	10	3358353	0.167	1.339E-06	583	C/G	H121D
C_FLESH	CmERF2ex2	MELO3C012242	10	1742964	0,149	7.931E-06	741	G/T	G171G
C_FLESH	MLO625760.1	MELO3C025760	11	22071980	0,095	0.0002283	384	A/G	Q128Q

Results are shown for markers that are significantly associated with various fruit ripening and sugar content traits, based on a mixed linear model (MLM) analysis of association using TASSEL [77]. Bonferroni’s correction was applied and the R^2^ and p-values for each association are indicated.

Three SNPs located in different genomic regions, CMPSNP711, SlERF1 and SlERF3 (Additional file 2) were associated with sugar content (Table 1). These were also significant with GLM analysis (Additional file 10). Although initially selected as a reference marker [4], CMPSNP711 in LG I was found to be associated with soluble solids content (°Brix) in the COMAV trial. This SNP is a 3´-UTR mutation in the MELO3C021106 gene, which putatively encodes xyloglucan glycosyltransferase 6. The C allele is more common in accessions producing fruits with low sugar content. It is fixed in sub-populations 6 and 7, comprised of low sugar *conomon* and non-sweet African *agrestis* accessions. The alternative allele is fixed in populations 1, 2 and 3 (sweet *cantalupensis* and Spanish *inodorus*), except in the Apelsinaja cultivar from Russia, the accession with the lowest brix degree in sub-population 2. Sub-populations 4 and 5 and the admixture group, with variable sugar content, contained both alleles, with the C allele occurring more frequently in the less sweet genotypes (Additional file 9). CMPSNP711 is located in a more distant part of chromosome I than other sugar content QTLs that have been mapped to the same linkage group [11].

Two melon orthologs of tomato ethylene responsive factors genes, *SlERF3* and *SlERF1* [52], carrying synonymous A/G (Q138Q) and C/T (N62N) mutations, respectively, were also found to be significantly associated with sugar content (Table 1). The A allele of the SlERF3 SNP was fixed in the non-sweet African *agrestis* (sub-population 7), and frequent in the low sugar genotypes of the admixture group. The alternative G allele was fixed in the remaining populations, as defined by the STRUCTURE analysis, and correlates with higher °Brix content in the admixture group. This SNP is located in LG XII in a region in which QTLs for sugar content have been reported previously [11]. Interestingly, mutation of *SlERF1,* located in LG III was consistently associated with soluble solids content in both trials and to sucrose content. The C allele is more frequent in *conomon*, *cantalupensis* groups (both Charentais and *reticulatus*) and Spanish *inodorus,* while European and Asiatic *inodorus* and *ameri* share the alternative allele with *African agrestis* and most of the low sugar types of the admixture group (*acidulus*, *flexuosus*, *chate* and *momordica*) (Additional file 9). A hotspot of QTLs involved in sugar accumulation has been described previously on this region of LG III [11].

Fruit flavor is also affected by acidity. Malic acid content was significantly associated with CmINHLIKE2.2, CMPSNP677 and AtEIN3ex4 (Table 1, Additional file 9). The SNP in the invertase inhibitor gene *CmINHLIKE2* corresponds to a synonymous T/C (S60S) mutation. The C allele found in all *conomon* was also present in *flexuosus, chate*, *dudaim*, *momordica*, *acidulus* and some wild American and Indian *agrestis*, all accessions with acid pulp rich in malic acid. The 3´-UTR mutation in CMPSNP677 (MELO3C009586 encoding a melon orthologue of the Arabidopsis ubiquitin carboxyl-terminal hydrolase 12-like protein) was initially selected as a reference SNP, but was also found to be related to malic acid content. The allelic distribution was similar to that of CmINH-LIKE2.2, except that the *conomon* allele is shared not only with *flexuosus*, *dudaim*, *momordica*, *acidulus* and wild American and Indian *agrestis,* but also with African *agrestis*. QTLs for glucose, fructose and soluble solids have previously been located in the chromosomal region of LG IV where this gene is located [11]. Finally, a synonymous C/T mutation (AtEIN3ex4.2 F375F) in *AtEIN3* was also associated with malic acid content. For this SNP, the *conomon* allele is shared with *acidulus* and Indian and American wild types. In Arabidopsis, the *AtEIN3* transcription factor plays a role in ethylene signaling downstream of the ETR1 ethylene receptor, and is involved in fine tuning of the ethylene response [28]. The association observed in the present work suggests a potential role for this transcription factor in regulating organic acid content in fruit.

### Association analysis for fruit ripening

Similarly to what occurred with sugar related gene candidates, the GLM analysis showed associations with some ripening candidates. For example, a non-synonymous, tolerated mutation (H121D) in *CmXTH5* located in LG X, was associated with formation of an abscission layer and fruit detachment (Additional file 10). The alternative alleles of this gene are fixed in the highly climacteric and the non-climacteric *cantalupensis* and *inodorus*, as described above (Additional file 9). Furthermore *CmXTH5* was mapped in the same region as a QTL for fruit firmness on LG X [14].

MLM showed a significant association with the same trait of MLO65044.1, a synonymous C/T mutation (L454L) in an *MLO-like* gene [53] located in LG XII (Table 1). The homozygous CC genotype is found exclusively in most *inodorus* types of Spanish, Turkish or Portuguese origin, which are mostly non-climacteric, whereas most other accessions have a homozygous TT genotype, including both climacteric and non-climacteric types. No QTLs related to climacteric ripening or fruit abscission have been reported before in this chromosome region [11,17].

Fruit firmness is also related to ripening behavior. MLM found the marker PSI_41-B07 SNP (C/T, F167F), on LG XI, significantly associated with the variation in this trait. In this region a QTL for flesh firmness was previously reported [11]. The T allele is present in the great majority of the germplasm, while the C allele is characteristic of *conomon*, African and Indian wild *agrestis* and *acidulus*, which had the highest values for firmness.

### Association analysis for flesh color

Fruit flesh color is also related with both fruit quality and ripening behavior, as the flesh of most climacteric genotypes changes from green to orange during ripening. Flesh color is controlled by two major genes, *green flesh* (*gf*) and *white flesh* (*wf*), which interact epistatically [54] and give rise to orange, white or green flesh depending on the gene combination. The *gf* gene has been mapped to chromosome VIII [20,47], while *wf* is located on chromosome IX [17]. Nevertheless the variability in the intensity of flesh color suggests that the phenotypic influence of the two major genes is modulated by the action of other genes, and minor QTLs for color intensity have been mapped to chromosomes III, VI and VII [10,20]. MLM showed several mutations associated with the variation in the color parameter chroma C, the non-synonymous mutation in *CmXTH5*, the synonymous changes in *CmERF2ex2*, and MLO625760.1, a gene in LG XI that encodes an MLO-like protein (Table 1). The same region (16-27 cM) contains a SNP in a *ZEAXANTHIN EPOXIDASE* (*ZEP*) gene [11]. The results obtained here suggest a contribution from chromosome X and XI that is worth further investigation.

## Discussion

PCA and STRUCTURE analyses carried out in the current study, based on SNPs located in reference and candidate genes, provided a germplasm stratification and classification very similar to previous works based on other marker systems or un-selected SNPs [4,5,47]. Therefore, the variability in sugar- and ripening-related genes appears to reflect the known genetic variability of this species. This observation is supported by the fact that gene diversity at candidate genes is just slightly lower than in reference genes.

Our results confirm that the highest genetic variability among cultivated melons occurs in Western and Central Asia and in the Middle East, where a high degree of genetic admixture was found. This admixture is not unexpected in melon, as no major geophysical barriers are found in the geographical distribution of traditional melon varieties, so gene flow among different regions is not impeded. Asian varieties are thought to be the likely ancestors of the *cantalupensis* and *inodorus* group [5,55]. In fact, some known current cultivars and landraces from France, Spain, Italy, Israel, Japan, and USA that do not fit to the *cantalupensis* Charentais, *reticulatus* or *inodorus* morphotypes completely, show variable admixture degrees of several sub-populations.

Our results indicate that SNPs in ripening related candidates, both ethylene and cell wall related, have a higher weight than SNPs in sugar candidates on both sub-species and sub-population stratification. This might reflect the effect of human selection in the development of strictly non-climacteric and high climacteric cultivars within ssp. *melo*. In contrast, selection for strictly climacteric or non-climacteric ripening was probably less intense within the ssp. *agrestis* cultivars*,* which more frequently show an intermediate ripening behaviour. A number of consistent QTLs involved in climacteric ripening have been described previously [14,17,18,23]. However, QTLs for sugar content have been found less consistently [16,20,47], indicating a higher heritability of ripening-related QTLs. Traditional farmers might have exerted a stronger selection for ripening traits, selecting alleles underlying ripening phenotypic variability more efficiently and thereby fixing the alleles within a particular cultivar group.

Association of candidate SNPs with phenotypic variability has been investigated by two approaches: (i) by looking for alleles that are fixed or have a higher frequency within horticultural groups with characteristic phenotypic features, and (ii) by association analysis. Sugar content and ripening behavior were clearly related with genetic structure, so the use of MLM models to adjust for genetic structure would also reduce the power to detect associations. MLM analysis has resulted in a much smaller number of SNPs associated with the studied traits. This could be due to lack of linkage or to the association between genetic structure and trait variability, as alleles that are close to being fixed within groups could not be associated significantly after adjusting for population structure in the MLM analysis. Nevertheless, a consistent association of the SlERF1 SNP and sugar content has been found on LG III. This linkage group contains a sugar content QTL hotspot [11], and was also found to be significantly associated with sugar content in a previous study [56], providing good evidence that this region contains genes that influence sugar accumulation.

Allelic differentiation analysis provided information that is complementary to the association results. Some SNPs in sugar-related candidate genes appeared in genotypes with common features in sugar content. These include SNPs that could be related with the mechanisms that prevent sugar accumulation in African melons, such as SNPs in invertase inhibitor genes (*CmINH1* and *CmINHLIKE2*) and in other genes encoding enzymes or proteins involved in sugar metabolism (*CmFK3*, *CmVPELIKE2*, *CmSUS3* and *CmSPP1*). Also of interest are mutations with more balanced allele frequencies and those with differential patterns of variation within sugar accumulating and non-accumulating groups, such as the mutation in the acid invertase *CmAIN2* and other sugar-related genes (*CmVPELIKE3* or *CmAAG2)*. The fact that these three genes co-localize with previously reported QTLs for sugar content in LG IX and X [11] gives support to their role in sugar content variation. It is worth noting that the more permissive GLM analysis provided consistent association of SNPs in these three candidates (CmAIN2.3, CmINHLIKE 2.1 and CmVPELIKE3.2) with °Brix values in both locations and with sucrose content.

Both GLM and MLM gave significant associations of traits related with ripening behavior, such as the formation of the abscission layer and flesh firmness, with reference SNPs in genes located in LG XII and XI, the latter being co-localized with a QTL for flesh firmness [11] (Table 1 and Additional file 10). However, association of ripening-related candidate genes with these traits was only detected by GLM. For example, the mutation in *CmXTH5*, a xyloglucan endotransglycolase/hydrolase that contributes to xyloglucan depolymerization in ripening fruit, had contrasting genotypes in *cantalupensis* and *inodorus* groups and was consistently associated with the formation of abscission layer and fruit detachment. The co-localization of this gene with a QTL for fruit firmness in LG X [14] provides further evidence that *CmXTH5* could be a candidate gene for the QTL. Other candidate regions found to be associated to the formation of the abscission layer with GLM mapped in regions of LG II, III, and VI that carry QTLs involved in climacteric ripening [11,23]. In addition, ethylene pathway candidate genes (*ACS1, ETR1, AtEIN3*), that map to regions in which QTLs for ripening in LG III, V and VII have been previously reported, were found to be associated with fruit firmness. However, further studies are needed to see if these mutations affect gene expression or gene function, as this differential allelic distribution might also be an effect of the population structure.

Our study shows that association and allelic differentiation analysis could be used as complementary approaches in highly structured populations, such as the current one, in order to define candidate genes. A highly variable germplasm collection with low genetic structure would be necessary to increase the power of association studies in melon. The *ameri* group showed both high phenotypic and genotypic diversity. Therefore, cultivars from this group, along with cultivars from related admixture groups from the Near-Middle East region, would provide a suitable genotype set for association analysis in melon.

## Conclusions

The analysis of SNPs in sugar metabolism and ripening-related genes, and also in reference genes, has revealed differences in the amount of genetic diversity among the main groups. Ripening-related genes seem to contribute more to this structure than sugar metabolism related genes. We found specific alleles of candidate genes fixed in cultivar groups concomitant with differences in sugar or ripening behavior. This could be due to ancient selection of these alleles by early farmers, but given the strong genetic structure, it is not currently possible to distinguish between direct selection or genetic drift. Nevertheless, some SNPs were still found to be associated with ripening behavior or sugar accumulation even after taking the genetic structure of the collection into consideration. As LD is extremely low in melon germplasm, these SNPs should be tightly linked to the causal SNPs. Taking into consideration the low LD, the strong genetic structure of melon germplasm, and the relatively high phenotypic and genotypic diversity found among accessions of Near-Middle East origin within the admixture group, it should be feasible to define a panel of accessions derived from this group that has minimal genetic structure but retains sufficient genetic diversity for association analysis after more extensive genotyping.

## Methods

### Plant material

The panel of melon genotypes used in this study consisted of 175 accessions of diverse origins (Additional file 1), with all of the major types identified by Pitrat [3] within the two subspecies, *C. melo* ssp. *melo* and *C. melo* ssp. *agrestis,* being represented by multiple accessions. This was based on a previously assembled core germplasm collection [4,57], supplemented by germplasm from the Cucurbit Breeding group at the Institute for the Conservation and Breeding of Agrobiodiversity (COMAV) in Valencia and is currently conserved at the COMAV genebank (www.comav.upv.es). Subspecies *melo* accessions included the commercially important groups *inodorus*, *cantalupensis* and *reticulatus*. The *inodorus* group contains commercial cultivars, Spanish landraces and traditional cultivars from Southern and Eastern Europe, North Africa and Asia. The *cantalupensis* group includes accessions widely grown in Europe, such as ‘Charentais’ types and other French, American, Israeli and Japanese cantaloupes, commercial *reticulatus* varieties and other *cantalupensis* types. The rest of the ssp. *melo* accessions belong to *ameri, chandalak, adana*, *flexuosus*, *chate* and *dudaim* groups from Southern and Eastern Europe, Northern Africa, Central Asia and India. The *agrestis* subspecies was represented mostly by accessions of Asian and African origin*. Momordica* from India, *conomon, makuwa* and *chinensis* from Far-East Asia, and Indian wild melons together represented the Asian melon diversity. *Tibish* cultivars from Sudan, considered as the most primitive melon known, cultivated *acidulus* and wild melons from Africa were also included.

### SNP selection

Three collections of SNPs were selected: (i) reference (148 SNPs), (ii) sugar metabolism related (43 SNPs), and (iii) ripening related (including ethylene related elements as well as genes encoding enzymes for cell wall degradation) (60 SNPs) (Additional file 2). The reference SNPs were chosen to define the genetic structure of the collection, and were extracted from a larger SNP collection genotyped in a set of accessions that collectively represents the full range of variation within the species, most of which were included in the current study [4]. The criteria for reference SNPs selection were: (i) even distribution throughout the genome; and (ii) variation in the germplasm collection representative of the variability within the species (preferably with a major allele frequency (MAF) of less than 80%) [4].

The SNPs of the candidate genes were selected from a SNP collection generated after resequencing eight pools of accessions representing the main melon botanical groups [44], most of which are common with the collection described in [4] and are included in the current study. All are in the SNP collection in the Melogene database (www.melogene.net).

Candidate SNPs were selected from the full SNP collection by using the BLAST algorithm to search the sequences for candidate genes against the Melogene database. The genome position of candidate gene SNPs was defined according to melon genome assembly v3.5, and their position in the genetic map was also established relative to the mapped refrence SNPs, using the anchorage of the genome sequence to the genetic map available in the Melonomics database (www.melonomics.net) [4,45]. The following criteria were also used to select SNPs in candidate genes: (i) location in the coding region (preferably causing non-synonymous substitutions) or in the 5´- or 3´-untranslated regions (UTRs) of candidate genes; (ii) not linked to other SNPs (≥100-bp distance); (iii) not close to an intronic region; and (iv) preferably having a MAF of less than 80%.

Forty-three SNPs were located in nineteen candidate genes related to sugar metabolism. Thirteen of these candidate genes were selected from those reported in previous studies (acid α-galactosidases *CmAAG1* and *CmAAG2*, sucrose synthases *CmSUS2* and *CmSUS3*, sucrose P phosphatases *CmSPP1* and *CmSPP2*, fructokinase *CmFK3*, soluble acid invertase *CmAIN2*, cell wall acid invertases *CmCIN3* and *CmCIN4,* and invertase inhibitors *CmINH1*, *CmINH2* and *CmINH3*). Dai et al. [12] analyzed the expression of these candidate genes during fruit development and ripening in cv. ‘Dulce’, a sweet melon from the *reticulatus* group, using publicly available data from the ICUGI melon EST database (www.icugi.org). Some of them were mapped by Harel-Beja et al [16] and Diaz et al [11] and colocalize with QTLs associated with sugar content. Additional candidates were identified directly in the melon genome database according to their annotation (www.melonomics.net) (*CmAAG3*, *CmAAG4*, *CmINHLIKE2*, *CmVPELIKE* 1, 2 and 3 (Additional file 2).

Thirty four candidates involved in ethylene biosynthesis, signal reception and signaling, and cell wall disassembly were selected from the Melonomics database based on their genome annotation and information from previous studies in melon [58], tomato mutants [52], grapevine [59], *Arabidopsis thaliana* [60], apple [61] and peach [62] (Additional file 2). Ethylene receptors and signal transduction elements such as *CmETR1* [11] and the melon orthologs of the following genes were selected: (i) *PpERS1* from *Prunus persica* (*ERS1PRUPE*); (ii) *VvETR2, VvRTE1* and *VvCTR1* from *Vitis vinifera*; (iii) *AtCTR1* from *Arabidopsis thaliana*; and (iv) *SlETR3* from *Solanum lycopersicum. SlETR3 (NR)* was previously identified in the *NR* (*never ripe*) tomato mutant [63-66]. Furthermore, genes encoding components of the ethylene signalling pathway downstream of CTR1 were also analyzed. These included the melon orthologue of *AtEIN3*, other genes annotated as *EIN3* or *EIL (CmEIN3LIKE, CmEIN3LIKE2 and CmEIL3)* in the Melonomics database, and the melon orthologue of the *SlEBF* gene of tomato [67], which controls EIN3 degradation in the nucleus. Other candidates were regulatory elements that affect ACS activity *(CmE8, E4/E8, CmETO1LIKE* and the melon orthologue of *SlHB1*) [32,36], ethylene responsive factors (*CmERF1, CmERF2, CmERF3,* the melon othologue of the apple *MdERF2*, two melon orthologs of the tomato genes *SlERF1* and *SlERF3*) [14,52,62], and the melon orthologs of the tomato ripening mutants *Cnr* and *rin* [31,68]. Genes co-located with the major QTLs for ripening, or coding for proteins involved in ethylene synthesis and signaling, e.g. *SlSBP* and *CmETHIND* were also included in the SNP analysis, using information from melon genetic maps [11,14,15,23]. Finally, genes encoding proteins involved in cell wall degradation were considered, such as expasin3 (*CmEXP3*)*,* which has been hypothesized to contribute to hemicellulose depolymerization, and *CmXTH5,* which encodes a xyloglucan endotransglycolase/hydrolase that contributes to xyloglucan depolymerization in ripening fruit [69].

The effect of the variants was analyzed with SIFT (Sorting Intolerant from Tolerant, http://sift.jcvi.org/), which predicts whether an amino acid substitution will significantly affect protein function. Scores below 0.05 indicate a strong probability that protein function will be affected by the mutation [70] (Additional file 2).

### Sequenom MassARRAY® assay

Total DNA was extracted from each genotype in the collection, from young leaves, by the method described in Doyle and Doyle [71] with minor modifications [4]. To improve the quality of the obtained DNA, 70% ethanol containing 15 mM ammonium acetate was used in the final wash, and the DNA was treated with RNAse A. DNA concentrations (in TE buffer) were measured on an ABI7900 (Applied Biosystems) using PicoGreen fluorescent dye and adjusted to 50 ng/μl. SNP genotyping was performed using an iPLEX® Gold MassARRAY® Sequenom system at the eEpigenetic and Genotyping unit of the University of Valencia (Unitat Central d´Investigació en Medicina (UCIM), University of Valencia, Valencia, Spain).

### Melon fruit phenotyping

The whole collection was phenotyped in two locations. Three randomized plants per accession were grown in Valencia (Spain) (39°28′11″ N- 0°22′38″ W) at COMAV-UPV greenhouse facilities, and in VCO’s St Rémy station (France) (43°47′18″ N- 4°49′54″ E), during spring/summer 2008. The following fruit traits (one fruit per plant) were analyzed: (i) fruit weight; (ii) flesh color ( L*C*h*, lightness, chroma and hue angle were measured with a Minolta colorimeter, at COMAV facilities, while at VCO, color was scored visually on the following scale: 1 = green, 2 = green-white, 3 = green-yellow, 4 = white, 5 = cream, 6 = yellow, 7 = orange, 8 = orange-green, 9 = orange-white, 10 = other); (iii) total soluble solids, °Brix (measured from drops of juice using a Milwaukee MR32ATC refractometer); and (iv) flesh firmness (measured using a fruit pressure tester FT327 with a plunger diameter of 8 mm). Flesh color, firmness, and total soluble solids were measured at four points in the equatorial region of the mesocarp. Samples of flesh tissue from the same region were harvested for metabolite analysis from fruits grown at COMAV, immediately frozen in liquid nitrogen and stored at -80°C until analysis. Soluble sugars (glucose, fructose and sucrose) and malate were measured enzymatically in ethanolic extracts as described in Stitt et al. [72].

From the classical markers of climacteric ripening (peak in ethylene production, degreening of the rind, aroma production, formation of an abscission layer and fruit detachment), formation of an abscission layer and fruit detachment were selected as the most amenable for high-throughput phenotyping to classify the accessions as climacteric or non-climacteric. Formation of an abscission layer was scored visually in the assay performed at COMAV-UPV on a scale from zero (no abscission layer) to four (fully formed abscission layer). Fruit detachment was scored visually in the assay performed at VCO on the scale 1 = absent, 2 = no slip, 3 = half slip, 4 = full slip.

### Genetic variability analysis

The genetic diversity of the germplasm collection as a whole was assessed by principal component analysis (PCA) using the ‘adegenet 1.4-0’ package in the R project for statistical computing (version R.14.1; www.r-project.org) [73,74]. The population genetic structure was also analyzed with STRUCTURE v.2.3.3 software [75]. Twenty independent runs for each K value ranging from 2 to 10 were performed with a burn-in length of 500,000 and 1 million iterations. The optimal subpopulation was calculated from the second order rate of change of likelihood (ΔK method) [48].

Basic genetic variability parameters, MAF, gene diversity (expected heterozygosity), observed heterozygosity (Ho) and Fisher’s fixation index (Fis) were calculated for each locus with PowerMarker 3.5 [76]. In order to investigate which SNPs could be responsible of the genetic structure, Fst and Analysis of Molecular Variance (AMOVA) were also studied for each SNP using the STRUCTURE defined groups with PowerMarker 3.5.

### LD estimation

TASSEL v 5.0 [77] (http://www.maizegenetics.net) was used to estimate the LD parameter r^2^ and the comparison-wise significance was computed from 1000 permutations. LD decay was drawn as a smooth line from r^2^ against distance in kb, fitting the data using a second-degree, locally-weighted scatterplot-smoothing LOESS [78], implemented in an Excel plug-in [79]. A critical value of r^2^ was derived from a distribution of the unlinked r^2^. The parametric 95th and 99th percentiles of the unlinked-r^2^ distribution were taken as a population specific critical value of r^2^.

### Association analysis

Association analysis was investigated by general linear (GLM) and mixed linear models (MLM) implemented in TASSEL v. 5.0. In order to control genetic structure effects, a kinship matrix was calculated using reference SNPs Associations were considered statistically significant for an experimental-wise threshold p < 0.05 after adjusting by the Bonferroni’s correction based on the total number of tested markers (p < 0.000333 for individual test).

## Additional files

Additional file 1:
**Melon germplasm analyzed in the current study.** A given number, code, country of origin and local/commercial name is given for each accession. Accessions are classified according to the *Cucumis melo* subspecies (ssp. *melo* and ssp. *agrestis*) and cultivar group (*cantalupensis, reticulatus, inodorus, ameri* group (including *ameri*, *adana* and *chandalack*), *flexuosus* (including *chate*) and *dudaim* within ssp. *melo* and *momordica*, *acidulus*, *conomon* (including *chinensis, conomon* and *makuwa*), *tibish, chito* and *agrestis*, within ssp. *agrestis*), and others i.e., classification not clear. Additional file 2:
**List of genes considered in the association study.** Their position in the genome and scaffold according to Garcia-Mas et al. [45], gene name and annotation according to Melogene (www.melogene.net) and Melonomics (www.melonomics.net), position in the genetic map according to Esteras et al. [4] are reported. AA represents the aminoacid change due to the SNP and the effect of the mutation screened by SIFT. Additional file 3;
**Estimated number of clusters obtained with STRUCTURE for K values from 1 to 10 using SNPs data for the all germplasm collection. a)** Graphical representation of estimated mean L (k) and b) its derivative statistics ∆K. The graph below was obtained excluding k = 2 in order to evidence the subpopulations present in the germplasm. c) Table summarizing parameters of STRUCTURE simulations performed for each present K: mean likelihoods of models, their standard deviations, ∆K. Additional file 4:
**Genetic diversity parameters present in each group defined from STRUCTURE analysis.** MAF indicates Major Alelle Frequency, gene diversity is the expected heterozygosity, Ho the observed heterozygosity, and Fis is Wright's fixation index. Additional file 5:
**Quantitative traits measured in the study for each accession (mean).** The collection was phenotyped for Brix degree (measured from drops of juice using a refractometer) and for flesh firmness (measured using a fruit pressure tester with a plunger diameter of 8 mm) in two locations, in Valencia (Spain) at COMAV facilities, and in VCO’s St Rémy station (France). Additional fruit traits were analyzed at COMAV: fruit weight, flesh colour (measured with a Minolta colorimeter,L*C*h*, lightness, chroma and hue angle), and the formation of an abscission layer (scored visually on a scale from zero, no abscission layer, to four, fully formed abscission layer). Samples of flesh tissue were harvested for metabolite analysis (glucose, fructose and sucrose and malate) from fruits grown at COMAV. Additional fruit traits were analyzed at VCO: fruit detachment (scored visually on the scale 1 = absent, 2 = no slip, 3 = half slip, 4 = full slip) and flesh color (scored visually on the scale 1 = green, 2 = green-white, 3 = green-yellow, 4 = white, 5 = cream, 6 = yellow, 7 = orange, 8 = orange-green, 9 = orange-white, 10 = other). The subspecies, the cultivar group and the population or population mixture, according to STRUCTURE results in Figure 2, are indicated for each accession. Additional file 6:
**Genetic Diversity parameters for the candidate genes for ripening and sugar accumulation as well as for referencel markers. **MAF indicates Major Alelle Frequency, gene diversity is the expected heterozygosity and Ho refers to observed heterozygosity. Additional file 7:
**Loading for PC1 and PC2 of candidate genes for ripening, including ethylene and cell wall related, (orange) and sugar accumulation (blue).**
Additional file 8:
**Contribution of the candidate genes (orange ripening, including ethylene and cell wall related, blue sugars) to the genetic differentiation among groups based on STRUCTURE analysis.** The SNPs with higher contributions are listed on the first column followed by the category candidate gene. Fst and percentage of variance (R2) among groups explained for each locus after AMOVA are depicted in the last columns. Additional file 9:
**Genotyping results for the polymorphic SNPs in the germplasm collection.** Colors indicate the 7 groups of STRUCTURE in Figure 2. Additional file 10:
**Significant markers associated to the trait of interest from GLM analysis by using TASSEL.** The analyzed traits are described in Additional file 5.
