# Supplementary material for: Variability of candidate genes, genetic structure and association with sugar accumulation and climacteric behavior in a broad germplasm collection of melon (Cucumis melo L.)
Source: BMC Genet. 2015 Mar 19;16:28. doi: 10.1186/s12863-015-0183-2 (PMC4380257; doi:10.1186/s12863-015-0183-2)
Supplement: Additional file 3; — Estimated number of clusters obtained with STRUCTURE for K values from 1 to 10 using SNPs data for the all germplasm collection. a) Graphical representation of estimated mean L (k) and b) its derivative statistics ∆K. The graph below was obtained excluding k = 2 in order to evidence the subpopulations present in the germplasm. c) Table summarizing parameters of STRUCTURE simulations performed for each present K: mean likelihoods of models, their standard deviations, ∆K. [file 12863_2015_183_MOESM3_ESM.pptx]

## Slide 1
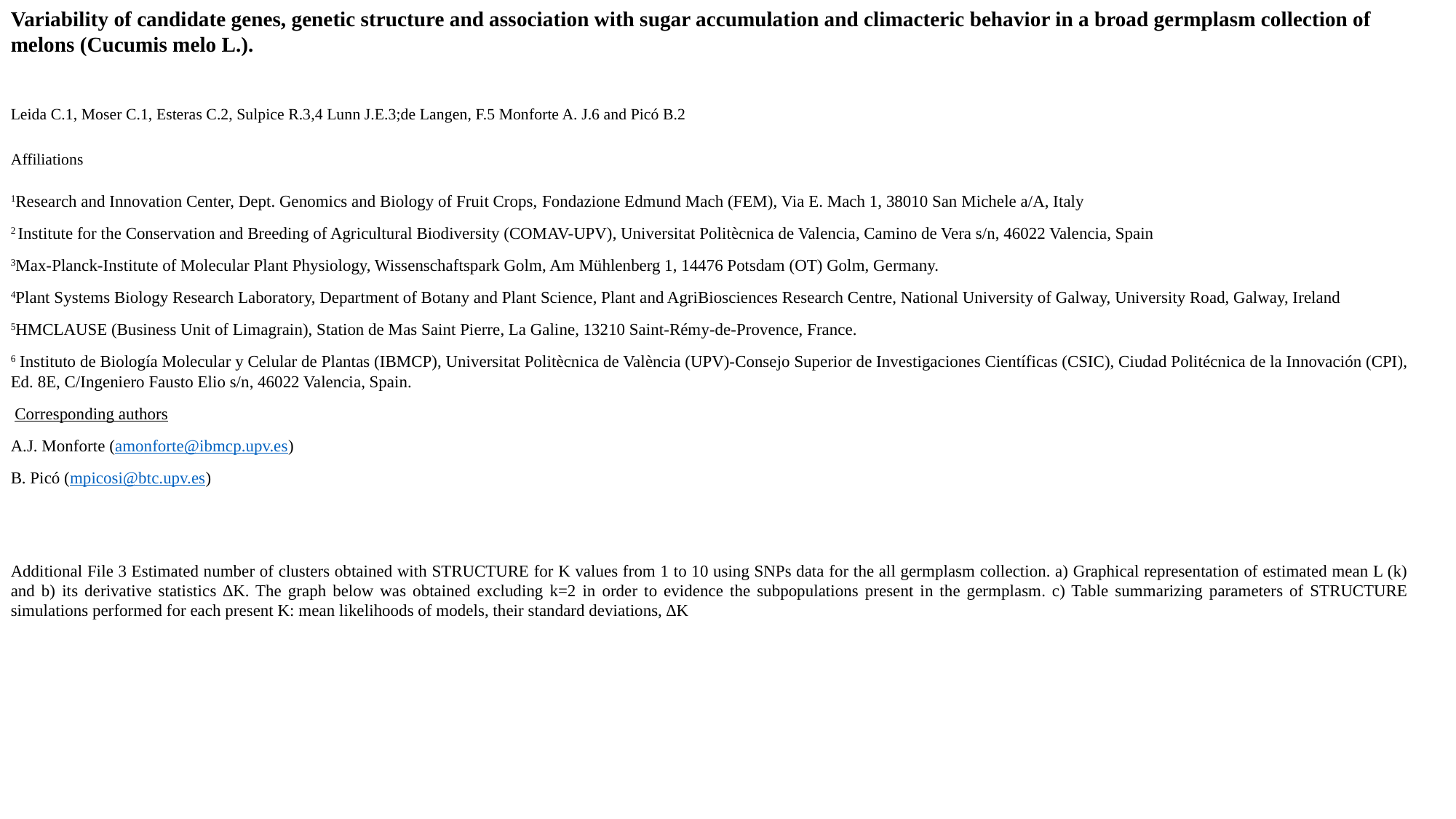

Variability of candidate genes, genetic structure and association with sugar accumulation and climacteric behavior in a broad germplasm collection of melons (Cucumis melo L.).
Leida C.1, Moser C.1, Esteras C.2, Sulpice R.3,4 Lunn J.E.3;de Langen, F.5 Monforte A. J.6 and Picó B.2
Affiliations
1Research and Innovation Center, Dept. Genomics and Biology of Fruit Crops, Fondazione Edmund Mach (FEM), Via E. Mach 1, 38010 San Michele a/A, Italy
2 Institute for the Conservation and Breeding of Agricultural Biodiversity (COMAV-UPV), Universitat Politècnica de Valencia, Camino de Vera s/n, 46022 Valencia, Spain
3Max-Planck-Institute of Molecular Plant Physiology, Wissenschaftspark Golm, Am Mühlenberg 1, 14476 Potsdam (OT) Golm, Germany.
4Plant Systems Biology Research Laboratory, Department of Botany and Plant Science, Plant and AgriBiosciences Research Centre, National University of Galway, University Road, Galway, Ireland
5HMCLAUSE (Business Unit of Limagrain), Station de Mas Saint Pierre, La Galine, 13210 Saint-Rémy-de-Provence, France.
6 Instituto de Biología Molecular y Celular de Plantas (IBMCP), Universitat Politècnica de València (UPV)-Consejo Superior de Investigaciones Científicas (CSIC), Ciudad Politécnica de la Innovación (CPI), Ed. 8E, C/Ingeniero Fausto Elio s/n, 46022 Valencia, Spain.
 Corresponding authors
A.J. Monforte (amonforte@ibmcp.upv.es)
B. Picó (mpicosi@btc.upv.es)
Additional File 3 Estimated number of clusters obtained with STRUCTURE for K values from 1 to 10 using SNPs data for the all germplasm collection. a) Graphical representation of estimated mean L (k) and b) its derivative statistics ∆K. The graph below was obtained excluding k=2 in order to evidence the subpopulations present in the germplasm. c) Table summarizing parameters of STRUCTURE simulations performed for each present K: mean likelihoods of models, their standard deviations, ∆K

## Slide 2
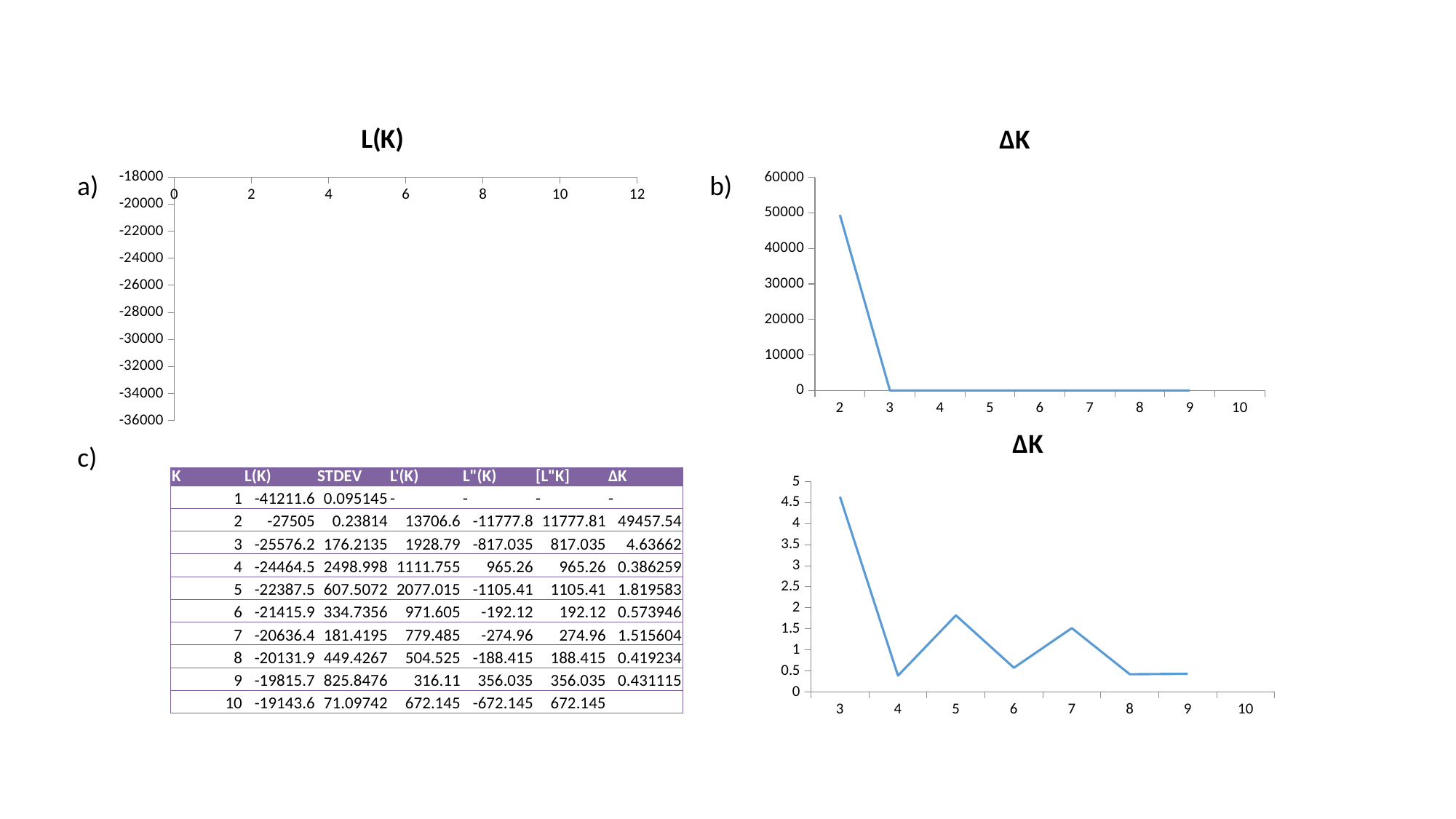

### Chart:
| Category | L(K) |
|---|---|
### Chart: ∆K
| Category | Delta K |
|---|---|
| 2 | 49457.54111 |
| 3 | 4.636619816 |
| 4 | 0.386258747 |
| 5 | 1.819583238 |
| 6 | 0.573945503 |
| 7 | 1.515603566 |
| 8 | 0.419234078 |
| 9 | 0.431114627 |
| 10 | None |a)
b)
### Chart: ∆K
| Category | Delta K |
|---|---|
| 3 | 4.636619816 |
| 4 | 0.386258747 |
| 5 | 1.819583238 |
| 6 | 0.573945503 |
| 7 | 1.515603566 |
| 8 | 0.419234078 |
| 9 | 0.431114627 |
| 10 | None |c)
| K | L(K) | STDEV | L'(K) | L"(K) | [L"K] | ∆K |
| --- | --- | --- | --- | --- | --- | --- |
| 1 | -41211.6 | 0.095145 | - | - | - | - |
| 2 | -27505 | 0.23814 | 13706.6 | -11777.8 | 11777.81 | 49457.54 |
| 3 | -25576.2 | 176.2135 | 1928.79 | -817.035 | 817.035 | 4.63662 |
| 4 | -24464.5 | 2498.998 | 1111.755 | 965.26 | 965.26 | 0.386259 |
| 5 | -22387.5 | 607.5072 | 2077.015 | -1105.41 | 1105.41 | 1.819583 |
| 6 | -21415.9 | 334.7356 | 971.605 | -192.12 | 192.12 | 0.573946 |
| 7 | -20636.4 | 181.4195 | 779.485 | -274.96 | 274.96 | 1.515604 |
| 8 | -20131.9 | 449.4267 | 504.525 | -188.415 | 188.415 | 0.419234 |
| 9 | -19815.7 | 825.8476 | 316.11 | 356.035 | 356.035 | 0.431115 |
| 10 | -19143.6 | 71.09742 | 672.145 | -672.145 | 672.145 | |
